# A New Inhibitor of Apoptosis from Vaccinia Virus and Eukaryotes

**DOI:** 10.1371/journal.ppat.0030017

**Published:** 2007-02-23

**Authors:** Caroline Gubser, Daniele Bergamaschi, Michael Hollinshead, Xin Lu, Frank J. M van Kuppeveld, Geoffrey L Smith

**Affiliations:** 1 Department of Virology, Faculty of Medicine, Imperial College London, London, United Kingdom; 2 Ludwig Institute for Cancer Research, Faculty of Medicine, Imperial College London, London, United Kingdom; 3 Department of Medical Microbiology, Nijmegen Centre for Molecular Life Sciences, Radboud University Nijmegen Medical Centre, Nijmegen, The Netherlands; University of Texas Southwestern Medical Center, United States of America

## Abstract

A new apoptosis inhibitor is described from vaccinia virus, camelpox virus, and eukaryotic cells. The inhibitor is a hydrophobic, multiple transmembrane protein that is resident in the Golgi and is named GAAP (Golgi anti-apoptotic protein). Stable expression of both viral GAAP (v-GAAP) and human GAAP (h-GAAP), which is expressed in all human tissues tested, inhibited apoptosis induced by intrinsic and extrinsic apoptotic stimuli. Conversely, knockout of h-GAAP by siRNA induced cell death by apoptosis. v-GAAP and h-GAAP display overlapping functions as shown by the ability of v-GAAP to complement for the loss of h-GAAP. Lastly, deletion of the v-GAAP gene from vaccinia virus did not affect virus replication in cell culture, but affected virus virulence in a murine infection model. This study identifies a new regulator of cell death that is highly conserved in evolution from plants to insects, amphibians, mammals, and poxviruses.

## Author Summary

Apoptosis is a conserved and strictly regulated process of cell suicide that, among other things, can remove virus-infected cells. In turn, many viruses, including poxviruses, have evolved strategies to block apoptosis to keep cells alive until virus replication is completed. Here, a novel viral anti-apoptotic protein from vaccinia virus and camelpox virus, viral Golgi anti-apoptotic protein (v-GAAP), and its human counterpart, h-GAAP, are described. Evolutionarily, GAAPs are extremely well conserved with closely related proteins in plants, insects, amphibia, and mammals, the viral and human counterparts sharing a striking 73% sequence identity. GAAPs are resident in the Golgi and inhibit apoptosis induced by a wide range of apoptotic stimuli. Knockout of h-GAAP, which is expressed in every tissue tested, induced cell death by apoptosis. The close relationship between the viral and the human proteins was confirmed in that v-GAAP could complement for the loss of h-GAAP and promote cell survival. Deletion of the v-GAAP gene from vaccinia virus affected virus virulence. Thus, this study identifies a new regulator of cell death that is highly conserved in evolution and has been hijacked by poxviruses. These data support a role for the Golgi complex in sensing pro-apoptotic stimuli.

## Introduction

Cell death is the result of either necrosis or apoptosis. Necrosis is the outcome of severe and acute injury, whereas apoptosis is an evolutionarily conserved, strictly regulated, energy-dependent process of cell suicide responsible for the ordered removal of superfluous, aged, or damaged cells, including virus-infected cells [1]. The signals that trigger apoptosis can be either extrinsic, and are sensed by death receptors at the cell surface, or intrinsic following perturbation of intracellular homeostasis, and are sensed by cellular organelles that initiate cell death [2]. The mitochondrion is the best known organelle regulating apoptosis, but the endoplasmic reticulum (ER), lysosomes, nucleus, and Golgi apparatus can all transmit stress signals to mitochondria, and thus contribute to the induction of apoptosis [3–5].

Poxviruses are a family of large DNA viruses that replicate in the cytoplasm [6]. The *Orthopoxvirus* genus is the most extensively characterised genus of the Chordopoxvirinae [7] and includes variola virus*,* the cause of smallpox, vaccinia virus (VACV), the vaccine used to eradicate smallpox, and camelpox virus (CMLV), which is closely related genetically to variola virus [8]. The orthopoxviruses have genomes of approximately 200 kilobase pairs and encode about 200 proteins [9]. Proteins encoded in the terminal regions of the genome are mostly non-essential for virus replication but affect virus virulence, host range, or the host responses to infection. This group of proteins is numerous and includes those that are secreted from the infected cell and bind complement factors, interferons, cytokines, and chemokines [10]. Alternatively, poxvirus immunomodulatory proteins may be intracellular and interfere with signalling pathways regulating cellular gene expression or apoptosis. Several poxvirus proteins that inhibit apoptosis have been described (for review, see [11]).

This study describes the identification and characterisation of a new poxvirus anti-apoptotic protein that is located in the Golgi and is called viral Golgi anti-apoptotic protein (v-GAAP). v-GAAP is non-essential for virus replication in cell culture but affects virus virulence in a murine model of infection. Strikingly, v-GAAP shows very high conservation with a hitherto uncharacterised human protein (h-GAAP) that is expressed in all human tissues tested, is essential for cell viability, is anti-apoptotic, and is conserved in plants, insects, amphibia, and mammals.

## Results

### Identification of GAAPs

**Figure 1:**
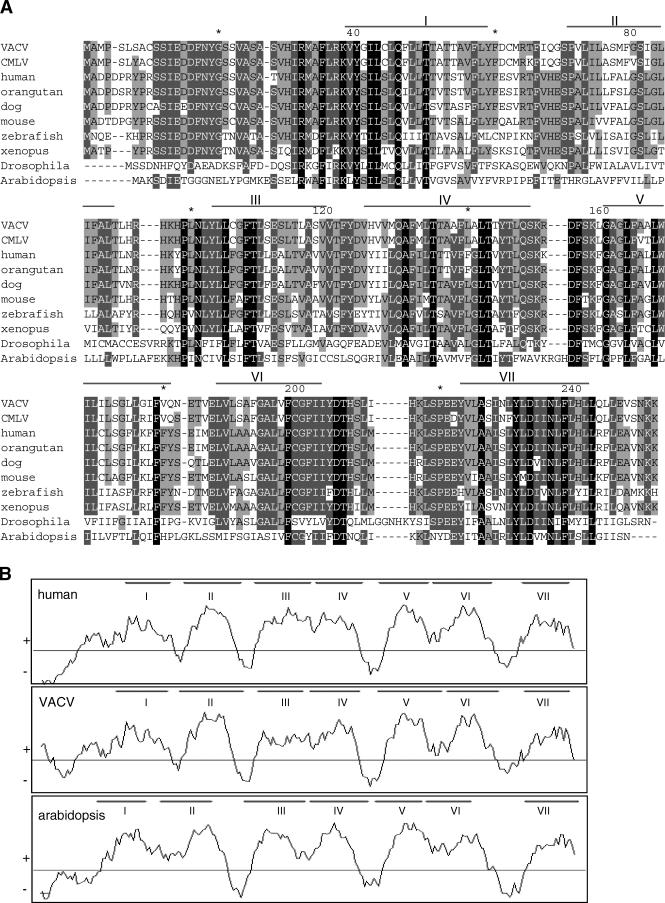
GAAPs Are Evolutionarily Conserved (A) CLUSTALW [47] alignment of h-GAAP and v-GAAP with related proteins from other species: VACV strain Evans, CMLV strain CM-S, human, orangutan, dog, mouse, *X. laevis,* zebrafish, *A. thaliana,* and D. melanogaster. Identical residues are shown in black, and residues conserved in six to eight or nine out of ten sequences are in light or dark grey, respectively. Horizontal bars show predicted transmembrane domains (I–VII) based on the Kyte–Doolittle hydropathy model. An asterisk denotes a block of 20 aa. (B) Hydrophobicity profiles of human, VACV, and A. thaliana GAAPs according to Kyte and Doolittle, plotted using the program WinPep [48]. Putative transmembrane domains (I–VII) are outlined.

CMLV gene *6L* encodes a 237–amino acid (aa) protein with a putative molecular mass of 26.5 kDa [8]. Database searches revealed a striking similarity to a human protein (73% identity, h-GAAP) of unknown function that is present in public databases under different names (e.g., z-protein, CGI-119, and S1R protein). BLAST searches with h-GAAP identified closely related, uncharacterised proteins in orangutan (96% identity), dog (89% identity), mouse (84% identity), rat (84% identity), Xenopus laevis (69% identity), and zebrafish (65% identity). In addition, there are related proteins of similar size predicted to be encoded by Arabidopsis thaliana (38% identity) and Drosophila melanogaster (34% identity) (Figure 1A).

Comparison of the sequence of all putative GAAPs showed that, evolutionarily, GAAPs are extremely well conserved in length (235–239 aa) and sequence identity. The close relationship between GAAPs is supported further by their conserved hydrophobicity profile, which predicted a six or seven transmembrane topology (Figure 1B and unpublished data). The PFAM program [12] assigned the human and viral GAAPs to the protein family UPF0005, whose members are predicted to contain six or seven transmembrane domains [13]. Two characterised UPF0005 family members show similarity to GAAPs. Bax inhibitor-1, which protects cells against ER stress, shares 28% identity (45% similarity) [14,15], and lifeguard, which protects cells from Fas-mediated cell death in the brain and other tissues, shares 34% identity (51% similarity) [16].

Collectively, these data suggest that GAAPs have an important and evolutionarily conserved function, possibly in regulating apoptosis.

### v-GAAP Is an Early Protein That Is Non-Essential for Virus Replication and Localises to the Golgi

**Figure 2:**
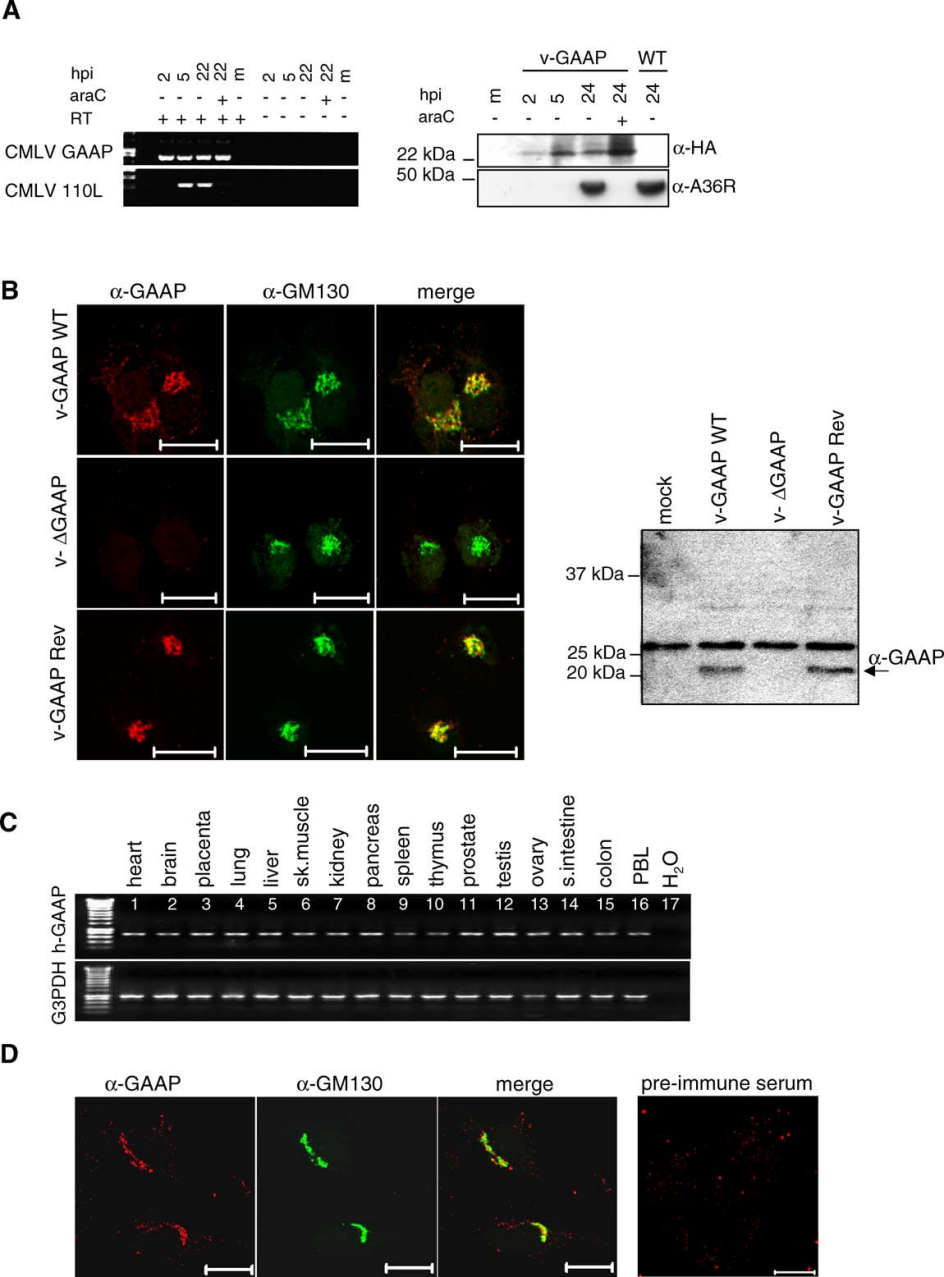
Human and Viral GAAPs Are Golgi Proteins (A) Time of v-GAAP expression during infection. Semi-quantitative RT-PCR (30 cycles) analysis of CMLV-infected TK^−^143 cells for presence of mRNA for v-GAAP and CMLV gene *110L* (equivalent to VACV gene *D8L*) (left panel). The right panel shows an immunoblot analysis of v-GAAP RevHA–infected TK^−^143 cells for expression of v-GAAP and the late VACV protein A36 using α-HA and α-A36 mAbs, respectively. m, mock. (B) v-GAAP localises to the Golgi. HeLa cells were infected with v-GAAP WT, v-ΔGAAP, and v-GAAP Rev for 6 h and stained for v-GAAP using anti-GAAP Ab and for Golgi using anti-GM130 Ab. Scale bars, 20 μm. The right panel shows an immunoblot analysis of v-GAAP mutant viruses 16 h p.i. using the α-GAAP Ab. The black arrow (left panel) indicates the GAAP protein. (C) Tissue expression profile of h-GAAP. The 714-bp *h-GAAP* gene (35 cycles, top panel) or 983-bp glyceraldehyde-3-phosphate dehydrogenase (G3PDH) gene fragment (30 cycles, bottom panel) were amplified by PCR from human cDNA master panels and analyzed by agarose gel electrophoresis. intestine, small intestine; PBL, peripheral blood leukocyte; sk. muscle, skeletal muscles. (D) h-GAAP localises to the Golgi. HeLa cells were stained for endogenous h-GAAP and Golgi marker GM130 using anti-GAAP and anti-GM130–specific Abs. Scale bars, 20 μm. Pre-immune serum was used as a control (right panel).

To examine the kinetics of v-GAAP protein expression, cells were infected with CMLV in the presence or absence of cytosine arabinoside (AraC), an inhibitor of DNA replication and late gene expression. Reverse transcription (RT)–PCR detected v-GAAP messenger RNA (mRNA) from 2 h post infection (p.i.) onward and at 22 h p.i. in the presence of AraC. In contrast, the CMLV late gene *110L,* a counterpart of VACV gene *D8L* [8,17], was only transcribed late during infection and not in the presence of AraC (Figure 2A, left panel).

There is no established small animal model for studying the contribution of specific genes to CMLV pathogenesis, so to investigate the role of v-GAAP in virus virulence, we searched other orthopoxviruses for orthologues of CMLV 6L. Bioinformatic analysis of poxvirus genomes (http://www.poxvirus.org) showed that the majority of orthopoxviruses do not encode an orthologue of CMLV gene *6L,* but an exception was VACV Lister [18]. PCR amplification of genomic DNA of VACV strains Lister, USSR, and Evans using primers for CMVL *6L* yielded a 714–base pair (bp) fragment, the same length as the CMVL *6L* gene (unpublished data). DNA sequencing confirmed that these viruses encoded proteins of 237 aa with 94%–95% identity with CMVL 6L.

To study the role of the viral protein, a set of VACV strain Evans viruses (v-GAAP WT) were constructed in which the *v-GAAP* gene was deleted (v-ΔGAAP) and re-inserted (v-GAAP Rev) into the deletion mutant. We also constructed a virus in which v-GAAP contained a C-terminal hemagglutinin (HA) tag (vGAAP RevHA). PCR and Southern blot analysis confirmed that the genomic structures of all viruses were as predicted. Comparisons of the growth of the recombinant viruses in cell culture showed there were no differences in plaque phenotype or yield of intracellular virus (unpublished data). Immunoblotting of extracts from v-GAAP RevHA–infected cells with anti-HA antibody (Ab) detected v-GAAP as a 23-kDa protein that was present early and throughout infection (Figure 2A, right panel). In contrast, VACV protein A36 was expressed only late during infection as reported previously [19]. These analyses showed that the kinetics of expression of the CMLV and VACV *v-GAAP* genes were indistinguishable.

To study the intracellular location of GAAPs, an Ab was raised against a GAAP peptide, SSIEDDFNYGSSV (h-GAAP aa 11–23 and v-GAAP aa 10–22). Cells were infected with v-GAAP WT, v-ΔGAAP, and v-GAAP Rev and fixed for immunofluorescence analysis 3, 6, or 24 h p.i. (Figure 2B, left subpanels; unpublished data). v-GAAP co-localised with the Golgi marker GM130 at each time point. Note that under these conditions, endogenous h-GAAP was not detected due to its lower level of expression (Figure 2B, middle left subpanel). In addition, very late post-infection (24 h) v-GAAP was also detected in the nuclear membrane and the ER (unpublished data). The α-GAAP antibody also recognised v-GAAP as a 23-kDa protein in extracts of cells infected with v-GAAP WT and v-GAAP Rev but not v-ΔGAAP (Figure 2B, right panel).

Therefore, v-GAAP is expressed early in VACV infection, localises to the Golgi, and is dispensable for VACV growth in cell culture.

### h-GAAP Is Expressed in All Tissues Tested and Localises to the Golgi

GAAPs are extremely well conserved evolutionarily (Figure 1). To investigate whether h-GAAP was expressed in different human tissue, complementary DNA (cDNA) from 16 human tissues obtained from multiple disease-free individuals was used for PCR analysis of h-GAAP gene expression. This showed that h-GAAP is expressed in all human tissue examined (Figure 2C). Next, the α-GAAP Ab was used to study the location of h-GAAP by immunofluorescence; this demonstrated that endogenous h-GAAP co-localised with the Golgi marker GM130 in HeLa, SiHa, and BGMK cells (Figure 2D and unpublished data). Therefore, h-GAAP is widely expressed in human tissues and, like v-GAAP, is a Golgi protein.

### Expression of GAAPs

**Figure 3:**
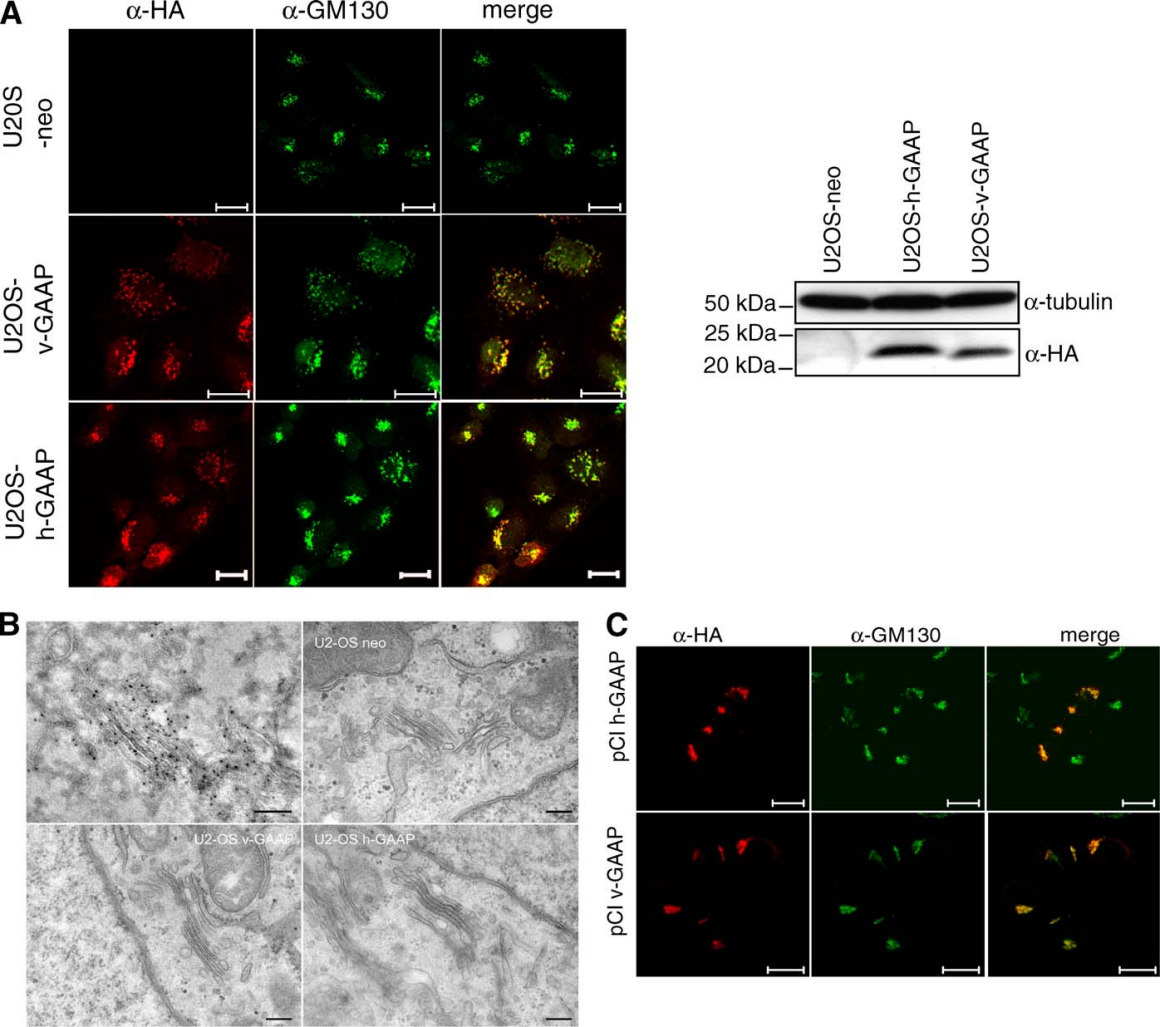
Location of GAAPs (A) U2OS-neo (top row), U2OS-v-GAAP (middle row), or U2OS-h-GAAP (lower row) cells were stained using an anti-HA mAb together with an α-GM130 Ab. All primary Abs were detected with secondary Abs conjugated to fluorescein isothiocyanate or tetramethylrhodamine isothiocyanate. Scale bars, 20 μm. The right panel shows an immunoblot analysis of U2OS stable cell lines using an α-HA mAb. (B) Top left panel: Cryo-immunoelectron microscopy was used to label the v-GAAP HA-tagged protein in U2OS v-GAAP cells with anti-HA mAb (Covance, diluted 1/10), followed by rabbit anti-mouse (Cappel) and 6-nm protein-A Au (scale bars 100 nm, top left panel). Golgi stack morphology was compared in the different cell lines by conventional thin sections of Epon-embedded samples and was examined by electron microscopy in U2OS-neo (top right panel), US-OS-v-GAAP (lower left panel), and U2OS h-GAAP (lower right panel). Scale bars, 200 nm. (C) HeLa cells were transfected with pCI-h-GAAPHA or pCI-v-GAAPHA and fixed 4 h post transfection. GAAPs were detected using an α-HA mAb, and cells were co-stained with the Golgi marker α-GM130. Scale bars, 20 μm.

To investigate the function of GAAPs, we expressed h-GAAP or v-GAAP bearing a C-terminal HA tag in stable cell lines (U2OS-v-GAAP and U2OS-h-GAAP) (Figure 3A and 3B) and in cells transiently by transfection (Figure 3C). In U2OS cells stably expressing h-GAAP or v-GAAP, the expression levels varied among individual cells, but, in all cases, GAAPs co-localised with the Golgi markers GM130 (Figure 3A) and giantin (unpublished data). This subcellular distribution was the same as for v-GAAP during VACV infection (Figure 2). In cells with higher expression levels, h-GAAP and v-GAAP were also seen in a reticular network characteristic of the ER (co-staining with protein disulfide isomerase, unpublished data). A similar distribution was seen in transfected cells (Figure 3C): early after transfection, viral GAAPs localized to only the Golgi (Figure 3C), but later, as their concentration increased, they were also present in the ER (unpublished data). To examine if GAAP expression was polarised to either the *cis* or *trans* Golgi cisternae, U2OS-v-GAAP and U2OS-h-GAAP cells were analysed by immunoelectron microscopy using protein-A gold (Figure 3B) or immunocytochemistry (unpublished data). v-GAAP was expressed throughout the stacks of the Golgi complex (Figure 3B, upper left panel), and similar results were obtained for h-GAAP (unpublished data). In U2OS-v-GAAP, and to a lesser extent in U2OS-h-GAAP cells, the Golgi complex appeared more dispersed than in parental U2OS cells (Figure 3A), but electron microscopy showed retention of Golgi stack morphology (Figure 3B). Moreover, transfection of a plasmid expressing vesicular stomatitis virus glycoprotein fused to green fluorescent protein (VSV-GFP) into these cells showed that protein trafficking to the cell surface was normal (Figure S1).

Therefore, both human and viral GAAPs are distributed throughout the Golgi stacks, and when they are overexpressed, glycoprotein trafficking to the plasma membrane is normal.

### h-GAAP Is Necessary for Cell Survival

**Figure 4:**
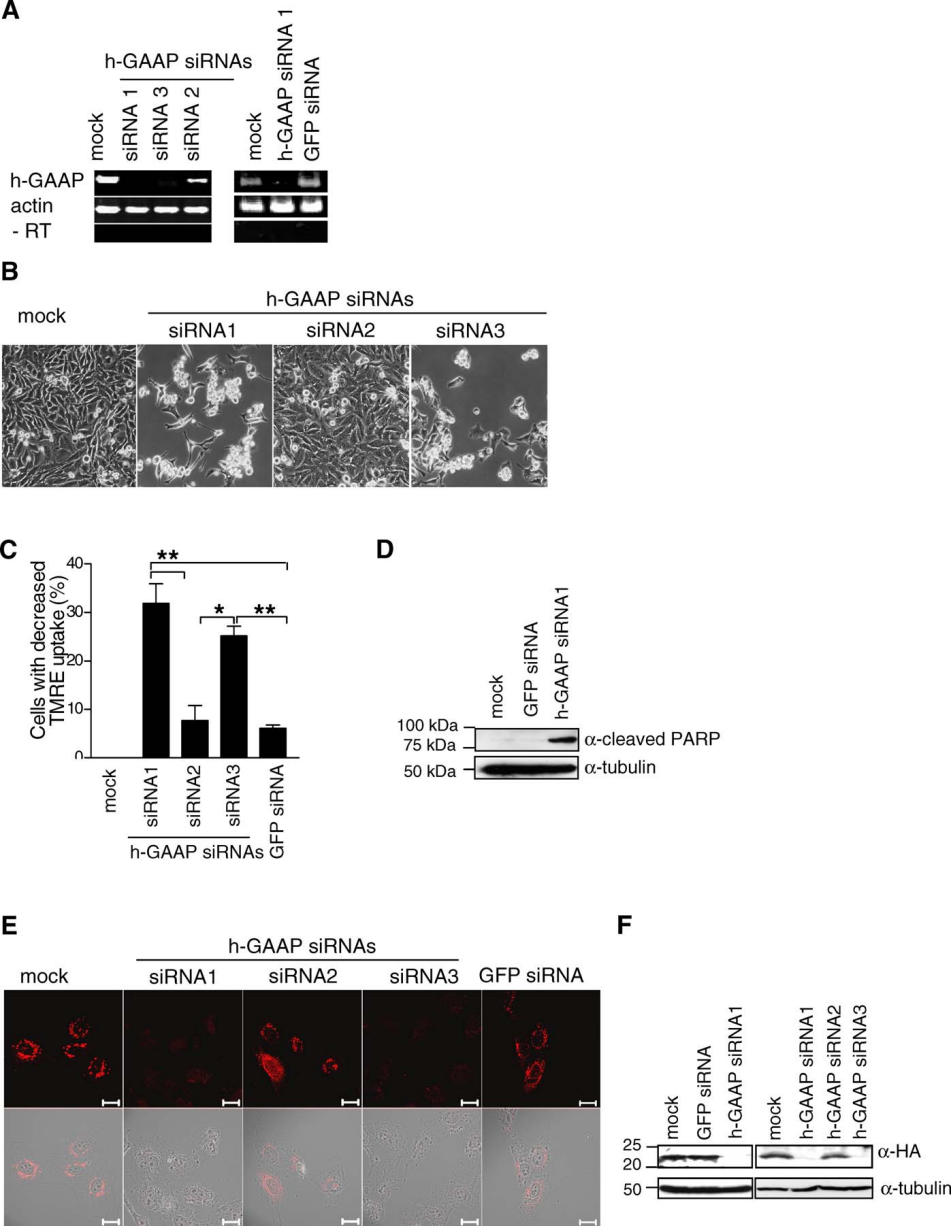
Downregulation of h-GAAP Causes Cell Death by Appotosis HeLa cells (A–D) or U2OS-h-GAAP cells (E and F) were transfected with siRNA oligos directed to h-GAAP (siRNAs 1–3) or GFP (GFP-siRNA) for 36 h (A and E), or 56 h (B–D and F). (A) mRNA level of h-GAAP in HeLa cells was assessed by semi-quantitative RT-PCR (35 cycles) using h-GAAP- or actin-specific primers, respectively. −RT indicates negative control. (B) HeLa cell morphology was recorded using a Zeiss Axiovert 200M Live Imaging System with an AxioCam HRc CCD. Images shown are representative of at least three independent experiments. (C) HeLa cells were loaded with the fluorescent dye TMRE and loss of the inner mitochondrial membrane potential was assessed by TMRE fluorescence two-color flow cytometry. The graph shows the percentage of cells with decreased TMRE uptake compared to mock-transfected cells. Data are mean percentages from at least three independent experiments (one of which is shown in Figure S2). *, *p* < 0.05; ** *p* < 0.005. (D) HeLa cells were treated with GFP-siRNA or h-GAAP siRNA1 or were mock-treated and assessed for cleavage of PARP by immunoblotting. (E and F) h-GAAP protein expression in U2OS-h-GAAP cells was assessed by immunofluorescence (E) or immunoblotting (F) using an α-HA mAb. In (E), the upper row of panels show the fluorescent images, and the bottom row shows a merged image of the top panels and an image of the same cells taken by differential interference contrast microscopy. Data are representative of three independent experiments. Scale bars, 20 μm.

To study h-GAAP function, three small interfering RNAs (GAAP siRNAs 1–3) were designed to down-regulate h-GAAP expression. RT-PCR showed that GAAP siRNAs 1 and 3 down-regulated h-GAAP mRNA substantially by 36 h post transfection, whereas GAAP siRNA2 and a control siRNA against GFP did not (Figure 4A). Strikingly, transfection of HeLa cells with GAAP siRNAs 1 and 3, but not GAAP siRNA2, induced cell death by 56 h post transfection (Figure 4B). Next, we measured the mitochondrial membrane potential using the membrane potential–sensitive dye tetramethylrhodamine ethyl ester (TMRE) 56 h after transfection of HeLa cells with GAAP siRNAs 1–3. Consistent with cell morphology changes, HeLa cells transfected with GAAP siRNAs 1 and 3 showed a decreased uptake of the TMRE compared with that of mock-transfected cells and cells transfected with GAAP siRNA2 and GFP-siRNA (Figures 4C and S2). In addition, immunoblotting showed cleavage of poly (adenosine diphosphate–ribose) polymerase (PARP) in HeLa cells transfected with h-GAAP siRNA1, but not in mock- or GFP-siRNA–transfected cells (Figure 4D). Immunofluorescence using U2OS-h-GAAP cells confirmed that h-GAAP protein expression was reduced to background by 36 h after transfection with GAAP siRNAs 1 and 3, but not with siRNA2 or GFP-siRNA (Figure 4E). Immunoblotting demonstrated that h-GAAP protein expression was undetectable 56 h after transfection with GAAP siRNAs 1 and 3, but not with siRNA2 and GFP-siRNA (Figure 4F). These data indicate that h-GAAP is necessary for cell survival.

### GAAPs Inhibit Apoptosis Triggered by the Intrinsic Pathway

**Figure 5:**
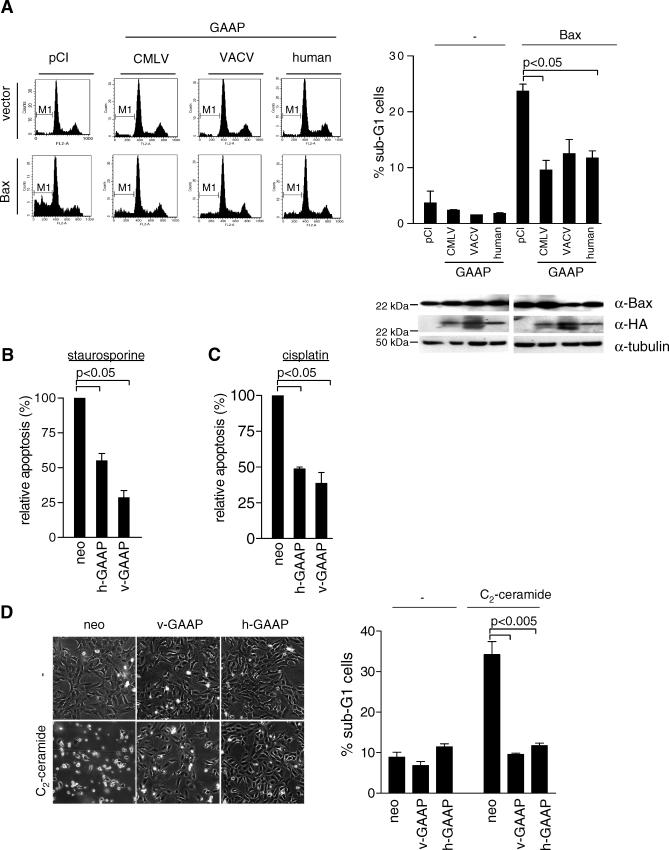
h-GAAP Decreases Cell Susceptibility to Intrinsic Apoptosis Pathways (A) Transient assay of Bax-induced apoptosis. Saos-2 cells were transfected with different plasmids as indicated, and representative flow cytometry profiles of CD20-positive cells are shown. The bar graph represents the percentage of sub-G1 cells (labeled M1 in the left panel). Data are mean percentages from at least two independent experiments. Protein expression was detected by immunoblotting using α-Bax or α-HA antibodies, respectively. (B–D) U2OS-neo, U2OS-v-GAAP, or U2OS-h-GAAP cells were mock-treated or treated with 3 μM staurosporine for 6 h (B), 10 μg/ml cisplatin for 24 h (C), or 10 μM C_2_-ceramide for 36 h (D), and apoptosis was assessed as above. The graphs show the relative percentage of apoptosis induced by staurosporine (B) or cisplatin (C). Data are mean percentages (± 1 standard deviation [SD]) from at least two independent experiments done in triplicate. (D) After 24 h treatment with C_2_-ceramide, the cell morphology was recorded as for Figure 3B. The graphs show the percentage of sub-G1 cells induced by C_2_-ceramide. Data are mean percentages (± 1 SD) from at least two independent experiments done in triplicate. Statistically significant differences are shown.

The similarity of GAAPs to anti-apoptotic proteins (lifeguard and Bax inhibitor-1) and the observation that knock down of h-GAAP expression induced apoptosis, suggest that GAAPs regulate apoptosis. Accordingly, we tested whether h-GAAP and v-GAAP affected the induction of apoptosis by five different pro-apoptotic stimuli that act through the intrinsic pathway (Bax, staurosporine, cisplatin, doxorubicin, and C_2_-ceramide) [20–24]. To test whether GAAPs modulate Bax-induced apoptosis, Saos-2 cells were transfected with an expression plasmid for Bax alone, or together with pCI-v-GAAP (CMLV or VACV Evans) or pCI-h-GAAP and the cell surface marker CD20. Figure 5A (left panel) shows representative DNA profiles of transfected (CD20-positive) cells 36 h post transfection. Quantification of sub-G1 cell populations (M1) showed that expression of Bax alone caused ~25% apoptosis among the transfected cell population, and this number was reduced by half when v-GAAPs (CMLV or VACV Evans) or h-GAAP were co-expressed with Bax (Figure 5A, right panel). Immunoblot analysis demonstrated that expression of GAAPs did not interfere with Bax expression levels (Figure 5B, right panel).

Apoptosis was then induced using staurosporine, a broad-spectrum kinase inhibitor. Staurosporine (3 μM) induced apoptosis in only 28% and 46% U2OS-v-GAAP and U2OS-h-GAAP cells, respectively, when compared to U2OS-neo cells (Figure 5B). To assess whether h-GAAP could also suppress cell death induced via p53 activation, U2OS-neo, U2OS-v-GAAP, and U2OS-h-GAAP cells were treated with cisplatin or doxorubicin. Treatment of cells with cisplatin (10 μg/ml) resulted in apoptosis in 38% and 48% of U2OS-v-GAAP and U2OS-h-GAAP cells, respectively, when compared to U2OS-neo cells (Figure 5B). Similar results were obtained after doxorubicin treatment (unpublished data).

Thus, GAAPs inhibit apoptosis in response to Bax, staurosporine, cisplatin, and doxorubicin. However, the most striking anti-apoptotic activity of GAAPs was against C_2_-ceramide (10 μM). This treatment induced apoptosis in 35% of U2OS-neo cells, whereas apoptosis in U2OS v-GAAP and U2OS-h-GAAP cells was inhibited completely and remained similar to untreated cells (Figure 5D).

### GAAPs Regulate Cell Susceptibility to Apoptosis Triggered by the Extrinsic Pathway

**Figure 6:**
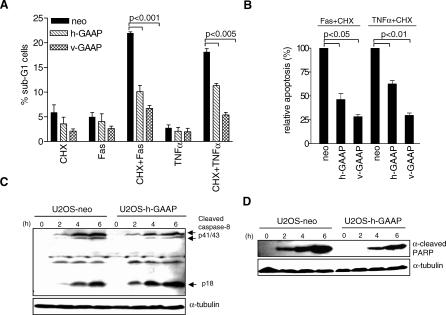
h-GAAP Decreases Cell Susceptibility to Extrinsic Apoptosis Pathways (A) U2OS-neo, U2OS-v-GAAP, or U2OS-h-GAAP cells were treated with 10 μg/ml of CHX alone or together with 500 ng/ml of anti-Fas Ab or 10 ng/ml of TNF-α. The bar graph represents the percentage of apoptotic cells (± 1 SD) after 24 h. (B) Relative inhibition of Fas and TNF-α–induced apoptosis in U2OS-neo (designated as 100%), U2OS-v-GAAP, and U2OS-h-GAAP cells. Data are mean percentages (± 1 SD) from at least three independent experiments. Statistically significant differences are shown. (C and D) After 2, 4, or 6 h incubation with TNF-α and cycloheximide, proteolytic cleavage of caspase-8 into p43/p41 and p18 fragments (C) and PARP (D) were assessed by immunoblotting.

To investigate whether h-GAAP and v-GAAP could inhibit apoptosis triggered via the extrinsic pathway, U2OS-neo, U2OS-v-GAAP, or U2OS-h-GAAP cells were treated with either anti-human Fas Ab (CH11) or human tumour necrosis factor (TNF)–α. Treatment with anti-Fas Ab and cycloheximide (CHX) caused apoptosis in 22% of U2OS-neo cells (Figure 6A) compared to 10% in U2OS-h-GAAP cells and 6% in U2OS-v-GAAP cells. Similarly, treatment with human TNF-α and CHX caused apoptosis in 19% of control cells, but only in 11% of U2OS-h-GAAP cells and 5% of U2OS-v-GAAP cells. Consistently (*n* = 3), compared to control cells treated with anti-Fas antibody or TNF-α (designated as 100%), apoptosis was reduced to 46.3% and 62.5% in U2OS h-GAAP cells and to 28.2% and 29.6% in U2-OS v-GAAP cells, respectively (Figure 6B).

To define the site of action of h-GAAP, we treated U2OS-neo and U2OS-h-GAAP cells with TNF-α and CHX and examined cleavage of caspase-8. There was no gross difference in proteolytic cleavage of caspase-8 into its active p43/p41 and p18 fragments between both cell lines after 2, 4, and 6 h (Figure 6C). In contrast, from 2 h onwards, cleavage of the PARP was reduced in U2OS-h-GAAP compared to that in U2OS-neo cells (Figure 6D). Similar results were obtained after Fas and CHX treatment (unpublished data) and when comparing U2OS-neo with U2OS-v-GAAP cells (unpublished data). Thus, overexpression of human and viral GAAPs provides partial protection against apoptosis triggered by the extrinsic pathway, and this inhibition takes place downstream of caspase-8 cleavage.

### v-GAAP Can Complement for Loss of h-GAAP

**Figure 7:**
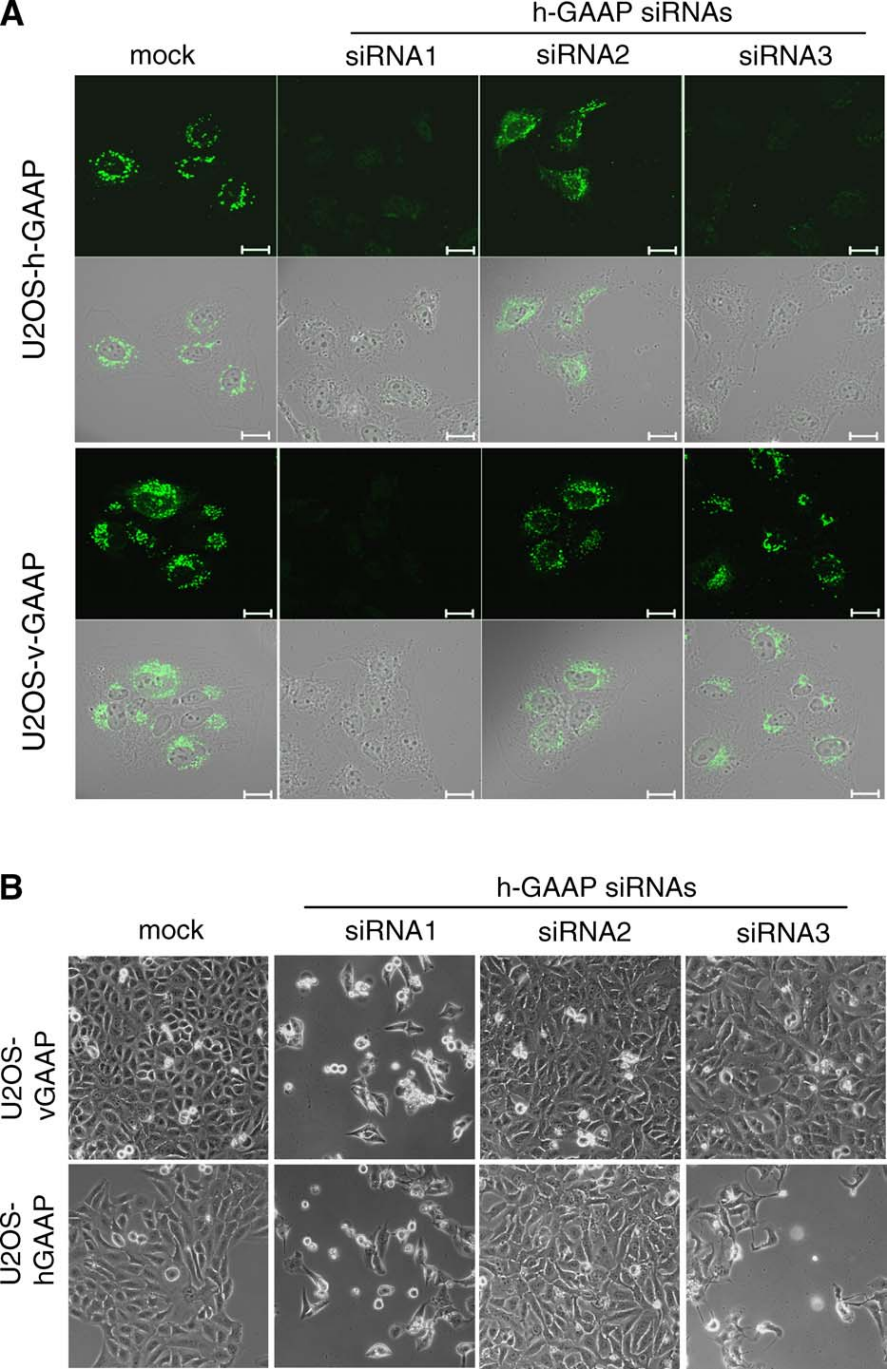
v-GAAP Complements for Loss of h-GAAP (A) U2OS-v-GAAP or U2OS-h-GAAP cells were transfected with siRNA oligonucleotides directed against h-GAAP (siRNAs 1–3) or v-GAAP (siRNA1) for 36 h. v-GAAP and h-GAAP expression was assessed by immunofluorescence using an α-HA mAb, followed by secondary Ab conjugated to FITC. The upper and third rows of panels show the fluorescent images, and the second and bottom rows show merged images of the fluorescent panel (immediately above) and an image of the same cells taken by differential interference contrast microscopy. Images shown are representative for at least three independent experiments. Scale bars, 20 μm. (B) Cell morphology of U2OS-h-GAAP or U2OS-v-GAAP cells 56 h after transfection with GAAP siRNAs 1–3 was assessed as for Figure 4B.

Human and viral GAAP RNA sequences share 77% nucleotide identity, so the use of specific siRNAs enables differential degradation of viral and/or human mRNA. GAAP siRNA1 was designed to down-regulate h-GAAP and v-GAAP, whereas GAAP siRNAs 2 and 3 were specific for h-GAAP. Accordingly, we tested GAAP siRNAs 1–3 for their ability to down-regulate v-GAAP in the U2OS human cell line that we had engineered to stably express v-GAAP, U2OS-v-GAAP. Immunofluorescence analysis showed that 36 h post transfection, GAAP siRNA1 down-regulated h-GAAP and v-GAAP, whereas siRNAs 2 and 3 did not affect v-GAAP expression, and siRNA2 affected neither h-GAAP nor v-GAAP (Figure 7A). Just as in HeLa cells (Figure 4B), U2OS-h-GAAP cells transfected with h-GAAP siRNAs 1 and 3, but not siRNA2, died by 48 h post transfection (Figure 7B, lower panels). In contrast, U2OS-v-GAAP cells, in which endogenous h-GAAP was down-regulated but v-GAAP was still expressed (siRNA3), remained healthy (Figure 7B, upper panels). Consistent with the cell morphology and the result of HeLa cell transfection (Figure 4B), U2OS-h-GAAP cells transfected with GAAP siRNAs 1 and 3 and U2OS-v-GAAP transfected with GAAP siRNA1 showed reduced uptake of TMRE (unpublished data).

Therefore, downregulation of h-GAAP causes cell death by apoptosis, and v-GAAP can complement for loss of h-GAAP to promote cell survival.

### v-GAAP Is a Virulence Factor

**Figure 8:**
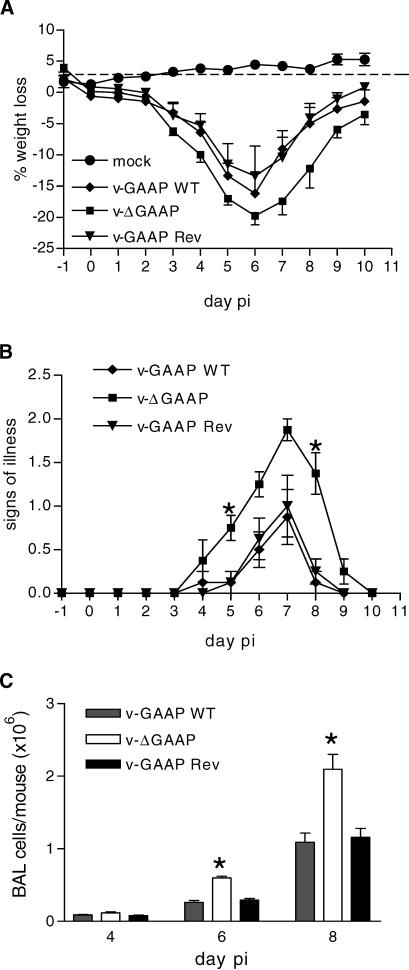
v-GAAP Is an Immunomodulator Groups of four BALB/c mice aged between 6 and 8 wk were mock-infected with PBS (•) or infected with 10^7^ pfu of v-GAAP WT (♦), v-ΔGAAP (), or v-GAAP Rev (▴). (A) Mice were weighed daily, and results are the mean percentage weight change of each group ± standard error of the mean (SEM) compared with the weight on the day of infection. (B) Animals from (A) were monitored daily for signs of illness, which was scored as described [27]. Data are expressed as the mean ± SEM. *p*-Values were determined by using Student's *t*-test and indicate mean weight loss (A) or mean signs of illness. *, *p*-values of mice infected with v-ΔGAAP that were significantly different from those of mice infected with v-GAAP WT and v-GAAP Rev (B). (C) Mice infected intranasally with 10^7^ pfu of v-GAAP WT, v-ΔGAAP, or v-GAAP Rev were sacrificed at various days p.i. as indicated. BAL cells were recovered and counted. Columns represent the mean cell yield per mouse ± SEM. Those marked with an asterisk indicate mean cell numbers recovered with v-ΔGAAP that were significantly different (*p* < 0.05) from those recovered from v-GAAP WT and v-GAAP Rev (three independent experiments with groups of four to five mice).

The role of v-GAAP protein in virus virulence was investigated using a murine intranasal model [25,26]. Mice were infected intranasally with 10^7^ plaque-forming units (pfu) of v-GAAP WT, v-ΔGAAP, or v-GAAP Rev, and the weight change and signs of illness (ruffled fur, arched backs, reduced mobility, or evidence of pneumonia [27]) were assessed daily. In repeated experiments (*n* = 5), there was no significant difference in weight loss when comparing mice infected with v-ΔGAAP to those infected with v-GAAP WT and v-GAAP Rev (Figure 8A). However, a significant increase in signs of illness (*p* ≤ 0.05, Student's *t*-test) was observed in v-ΔGAAP compared to both control groups on days 5 and 8 p.i. (Figure 8B). Similar results were obtained after infection of mice with 3 × 10^6^ pfu (unpublished data): mice infected with v-ΔGAAP showed increased signs of illness compared to mice infected with v-GAAP WT and v-GAAP Rev.

To assess why infection with v-ΔGAAP resulted in more severe signs of illness, the virus titres in lungs were determined at different times p.i. Each virus replicated well in lung tissue to reach titres >10^6^ pfu/gm of tissue by 3 d p.i., and there were no statistically significant differences between virus titres of the different groups detected on this day or later (unpublished data). Next, the inflammatory infiltrate in the lungs of infected mice was examined. In repeated experiments (*n* = 3), at days 6 and 8 p.i., significantly more cells were recovered from bronchoalveolar lavages (BALs) of mice infected with v-ΔGAAP than from mice infected with v-GAAP WT and v-GAAP Rev (Figure 8C; *p* ≤ 0.01, Student's *t*-test). As reported previously after VACV infection [28], there was a low level of cell recruitment to the lungs of infected animals early after infection, and this increased to a maximum at 10 d p.i. with v-GAAP WT and v-GAAP Rev, and at day 8 with v-ΔGAAP (unpublished data).

These results show that loss of v-GAAP altered the virulence of VACV in an intranasal murine model of infection.

## Discussion

A new regulator of apoptosis is described that is encoded by VACV, CMLV, and many other eukaryotes. This protein is called Golgi-anti-apoptotic protein because of its function and subcellular location. Overexpression of h-GAAP or v-GAAP rendered cells resistant to induction of apoptosis, while knock down of expression of the human gene by siRNA caused cells to die by apoptosis. Deletion of v-GAAP from VACV did not affect virus replication in cell culture, but affected virulence in a murine model of infection. v-GAAP therefore represents a new virus-encoded apoptosis inhibitor. v-GAAP is remarkable in that it is very closely related to a previously uncharacterised human protein (h-GAAP) that is conserved in a wide range of eukaryotes, is expressed in all 16 human tissues tested, and is essential for cell viability.

VACV and other poxviruses express a wide range of proteins that interfere with aspects of the host response to infection (for reviews see [10,29]). These proteins may act inside or outside the cell, and most of them target the innate response to infection. Among the intracellular regulators of the innate response, a subset block the induction of apoptosis, so that virus replication can be completed before the cells dies. Apoptosis regulators from VACV and other poxviruses have been reviewed recently [11] and include proteins that inhibit caspase activity, or are present at the mitochondrial membrane. v-GAAP is another example of an intracellular apoptosis inhibitor, but it has three striking differences from the ones discovered previously.

First, v-GAAP (and h-GAAP) is present in the Golgi, a compartment that until recently was not thought to have an important role in the regulation of apoptosis [5,30]. This observation supports recent evidence that the Golgi has a role in the regulation of apoptosis. Other poxvirus proteins that affect induction of apoptosis are located either at mitochondria or are in the cytosol.

Second, v-GAAP is very closely related to a human protein that shows remarkable conservation throughout eukaryotes. The virus and human protein have 73% aa identity, a similar hydropathy profile, and a very similar length. Moreover, the genomes of many eukaryotes (plants, insects, amphibia, and mammals) encode a closely related protein. For instance, the human and *Arabidopsis* GAAPs share 38% aa identity and differ in length by only 4 aa. Hitherto, the function of the protein we have called GAAP had not been determined in any species, although the human gene was one of several reported to be up-regulated in some breast tumours [31]. Data presented here provide the first characterisation to our knowledge of the human protein, and demonstrate that this protein is essential for cell viability and that cells die by apoptosis in its absence. In addition, it is shown that the viral protein can complement the essential function served by h-GAAP. Other immunoregulatory proteins from poxviruses either have no orthologues outside poxviruses or have a much lower amino acid identity with cellular counterparts. For instance, the interleukin-1β receptor [32], interferon-γ receptor [33], and 3β-hydroxy-steroid dehydrogenase [34] from VACV share 33%, 25%, and 44% aa identity with their human orthologues, respectively. Therefore, compared to other orthopoxvirus immunomodulators, v-GAAP shows by far the greatest amino acid similarity with its mammalian counterpart. The close relationship of the GAAPs in VACV and mammals suggests that either the gene has been captured from the host more recently in evolution, or that functional constraints have restricted its divergence after acquisition.

Third, v-GAAP (and h-GAAP) inhibits apoptosis induced by an unusually broad range of pro-apoptotic stimuli from both the extrinsic and intrinsic pathways. Instrinsic pro-apoptotic stimuli inhibited by GAAPs include bax, staurosporine, doxorybicin, cisplatin, and ceramide. Extrinsic stimuli inhibited include Fas and TNF. The mechanism by which GAAPs inhibit apoptosis remains to be determined. The ability of GAAPs to inhibit cell death induced by various instrinsic apoptotic stimuli suggests strongly that it acts at a step in the mitochondrial pathway. The finding that h-GAAP suppressed TNF-induced apoptosis at a step downstream of caspase-8 activation is consistent with this idea.

In summary, a new regulator of apoptosis has been discovered by the study of poxviruses. The protein, called v-GAAP in VACV and h-GAAP in *Homo sapiens,* is highly conserved in eukaryotes, and is another example of a host gene acquired during virus evolution to combat the host response to infection. The human protein is essential for cell viability, and in its absence cells die by apoptosis. Both human and viral GAAPs inhibit an unusually wide range of pro-apoptotic stimuli. The viral protein affects virus virulence and can also substitute for the h-GAAP to maintain cell viability.

## Materials and Methods

### Cells and viruses.

Monkey BS-C-1 and human HeLa, TK^−^143, U2OS (osteosarcoma, p53 wild-type), and Saos2 (osteosarcoma, p53-null) cells were grown in Dublbecco's modified Eagle's medium (Gibco, http://www.invitrogen.com) with 10% (v/v) heat-inactivated fetal bovine serum. The source of VACV strain Evans and CMLV strain CM-S were described previously [8,27].

### GAAPs antiserum.

The peptide sequence SSIEDDFNYGSSV (h-GAAP aa 11–23 and v-GAAP aa 10–22) was conjugated to keyhole limpet hemocyanin via a C-terminal cysteine residue and used to immunize rabbits (Sigma-Genosys, http://www.sigmaaldrich.com/Brands/Sigma_Genosys.html). The specificity of the Ab was confirmed by immunofluorescence using U2OS cells stably expressing HA-tagged human or viral GAAPs showing that the signal obtained is identical to the signal given by the anti-HA monoclonal antibody (mAb).

### Tissue expression profile of h-GAAP.

The presence of h-GAAP mRNA in different tissues was analyzed using Human Multiple Tissue cDNA panels I and II (Clontech, http://www.clontech.com) and semi-quantitative RT-PCR with Platinum PCR SuperMix (Invitrogen, http://www.invitrogen.com). cDNAs were amplified with h-GAAP specific primers. To define the optimal number of PCR cycles for linear amplification, an aliquot from each of the samples was removed at 20 cycles, and thereafter at every fifth additional cycle.

### Cloning of human and viral GAAPs.

CMLV strain CM-S and VACV strain Evans genomic DNA were used for PCR amplification of the *v-GAAP* genes with the influenza virus HA epitope YPYDVPDYA encoded at the 3′ end. These fragments were cloned into the plasmid pCDNA3.1 (Invitrogen). Oligonucleotides were identical for both viral proteins: v-GAAP exp1 (5′-GCCCCGAATTCCGCCGCC**ATG**GCAATGCCG) and v-GAAP exp2-HA (5′-GCCCCTCGAG **TAA**agcgtaatcaggcacgtcgtaagggtaTTTCTTATTAGATACTTCCAAAAGC). Bold, underlined, or lower case nucleotides represent the translation initiation and termination codons, restriction sites, and HA tag, respectively.

For the *h-GAAP* gene, total RNA was isolated from HeLa cells and h-GAAP cDNA was cloned by RT-PCR into pCDNA3.1 using the ThermoScript RT-PCR System (Invitrogen) and oligonucleotides h-GAAPexp1 (5′-GCCCCGAATTCTGCCATC**ATG**GCTGACCCCG) and h-GAAPexp2HA (5′- GCCCCTCGAG **TTA**agcgtaatcaggcacgtcgtaagggtaCTTTTTATTAACTGCTTCCAGAAACC). Bold, underlined, or lower case nucleotides represent the translation initiation and termination codons, restriction sites, and HA tag, respectively. The fidelity of all DNA sequences was confirmed by sequencing. For transient transfection experiments, human and viral GAAPs cDNA were subcloned into pCI (Promega, http://www.promega.com).

### Construction of GAAP mutant viruses.

A VACV mutant lacking the *GAAP* gene was constructed using a plasmid assembled by splicing by overlap extension (SOE)-PCR [35]. Two DNA fragments were amplified representing either the 5′ (307 bp) or 3′ (462 bp) flanking regions of the *GAAP* gene, using oligonucleotides ΔGAAP-1 (5′-GCCCCGTCGACGATGTGATAAATACAGCGATACC) and ΔGAAP-2 (5′-**CCAATCCATGTTT**TGACCTATATATTAATACTC) or oligonucleotides ΔGAAP-3 (5′-**TAATATATAGGTCA**AAACATGGATTGGAAAC) and ΔGAAP-4 (CGGGGAATTCGTAGCGTGGCAATGACAG), respectively. Annealing regions are shown in bold and restriction sites are underlined. The 5′ and 3′ GAAP deletion fragments were joined by SOE-PCR using oligonucleotides ΔGAAP-1 and ΔGAAP-4, and the product was cloned into pSJH7 [36], forming pSJH7-ΔGAAP.

To make a revertant virus, a wild-type copy of the *GAAP* gene was amplified by PCR using oligonucleotides ΔGAAP-1 and ΔGAAP-4 and VACV Evans genomic DNA as template. This product was cloned into pSJH7, forming pSJH7-GAAP-Rev. For the construction of a VACV expressing v-GAAP with a C-terminal HA tag, plasmid pSJH7-GAAPRevHA was constructed by SOE-PCR as for pSJH7-ΔGAAP using oligonucleotides ΔGAAP-1, ΔGAAP-2RevHA (5′-**AGCGTAATCAGGCACGTCGTA**AGGGTATTTCTTATTAGATACTTCCAAAAGC), ΔGAAP-3RevHA (5′- **TACGACGTGCCTGATTACGCT**TAAAGTTTAAAATAGAATTAATAAAAACATATAGG), and ΔGAAP-4 and VACV genomic DNA as template.

Recombinant viruses were constructed by transient dominant selection [6] using the Escherichia coli guanine xanthine phosphoribosyltransferase (Ecogpt) gene as the selectable marker [37]. Briefly, pSJH7-ΔGAAP was transfected into CV-1 cells that were infected with VACV Evans, and a deletion mutant (v-ΔGAAP) and a wild-type control virus (v-GAAP WT) derived from the same intermediate virus were isolated as described for VACV *A41L* gene [38]. A revertant virus with or without an HA tag (v-GAAP Rev and v-GAAP RevHA) were constructed in a similar manner, using VACV v-ΔGAAP as parent virus and plasmids pSJH7-GAAPRev and pSJH7-GAAPRevHA, respectively. All virus isolates were plaque-purified three times and the virus genomes were analysed by Southern blotting and PCR using DNA extracted from virus cores [39].

### Stable cell lines.

U2OS cells were transfected with either the pCDNA3.1- or pCDNA3.1-encoding human or viral GAAPs-HA using Lipofectamine (Invitrogen); 48 h post-transfection, cells were treated with 500 μg/ml of G418. After 1 mo, isolated colonies were cloned and the expression of transfected sequences was determined by immunofluorescence and immunoblotting. Staurosporine- and Fas-induced apoptosis assays (see apoptosis assay section below) were carried out with two independent U2OS-v-GAAP clones or three independent U2OS-h-GAAP clones. Each clone gave similar results to those shown for the two clones described throughout the paper.

### Flow cytometry.

Saos-2 cells (10^6^) were seeded in 10-cm diameter plates; after 24 h, cells were transfected with plasmids expressing CD20 (2 μg), Bax (5 μg), and 15 μg of pCI or pCI-v-GAAP or pCI-h-GAAP-HA as indicated. Then, 36 h after transfection, both attached and floating cells were harvested and analyzed as described [40].

### Apoptosis assays.

Cells were seeded into 6-well dishes at 4–6 × 10^5^ cells/well. One day later, cells were treated with 3 μM staurosporine, 10 mg/ml cisplatin, 500 ng/ml anti-Fas Ab (Clone CH11; Upstate Biotechnology, http://www.upstate.com), or 10 ng/ml of human TNF-α (PeproTech EC, http://www.peprotechec.com). Fas and TNF-α were used in combination with 10 μg/ml of CHX (Sigma). For induction of apoptosis by C_2_-ceramide (N-acetyl-D-sphingosine, Sigma), cells were preincubated in modified KRB buffer (125 mM NaCl, 5 mM KCl, 1 mM MgSO_4_, 1 mM Na_2_HPO_4_, 5.5 mM glucose, 20 mM NaHCO3, 2 mM L-glutamine, and 20 mM HEPES [pH7.4]) supplemented with 1 mM CaCl_2_ for 4 h and treated with 10 μM C_2_-ceramide in the same medium. At the indicated time post all treatments, both attached and floating cells were harvested using 4 mM EDTA/PBS. The DNA content of all the cells was analyzed using flow cytometry as described above.

### Immunofluorescence.

HeLa, U2OS-neo, U2OS-v-GAAP, or U2OS-h-GAAP cells were grown on coverslips, and intracellular proteins were stained as described [41]. For detection of endogenous h-GAAP, cells were fixed with 3% PFA in PBS on ice for 20 min, and permeabilised and stained as above. For detection of v-GAAP after VACV infection, HeLa cells were infected at 10 pfu/cell and fixed as above 4 h p.i. Samples were examined with a Zeiss LSM 510 laser scanning confocal microscope. Images were captured and processed using Zeiss LSM Image Browser version 3.1.0.99 (http://www.zeiss.com). Primary antibodies used were mouse α-HA mAb (1:1000; Covance, http://www.covance.com), rat high affinity α-HA mAb (clone 3F10, 1:1000; Roche, http://www.roche.com), rabbit α-GAAP (1:50), mouse α-GM130 (1:100, BD Transduction Laboratories; BD Biosciences, http://www.bdbiosciences.com), or mouse α-A36R mAb (1:100, [42]).

### Electron microscopy.

For conventional electron microscopy and for cryo-immunocytochemistry, cells were treated as described [43]. All images were acquired on a FEI Tecnai G2 electron microscope (http://www.fei.com) using a MegaView III CCD camera (Olympus Soft Imaging Solutions, http://www.soft-imaging.net) operating under analySIS Docu software.

### Immunoblotting.

Cells were lysed in RIPA or LDL buffer and between 50 and 200 μg of protein was analyzed by immunoblotting as described [41]. Primary antibodies used were rabbit α-GAAP (1:200), mouse α-HA mAb (1:1000; Covance), rat α-alpha tubulin (YL1/2, 1:2000; Serotec, http://www.ab-direct.com), and mouse α-cleaved PARP (Asp214) mAb (1:1000, 19F4; Cell Signaling Technology, http://www.cellsignal.com).

### RNAi transfection.

siRNA sequences specific to h-GAAP were designed using “siDIRECT” [44,45] and were from Ambion (http://www.ambion.com). GAAP siRNA1: 5′- CGAUCGAGGACGACUUCAACU-3′ (sense) and 5′- UUGAAGUCGUCCUCGAUCGAG-3′ (antisense); GAAP siRNA2: 5′- CUGUACGGACAUUUGUACAUG-3′ (sense) and 5′- UGUACAAAUGUCCGUACAGAC-3′ (antisense); GAAP siRNA3: 5′- CGGAUCUCUGGGUUUGAUUUU-3′ (sense) and 5′- AAUCAAACCCAGAGAUCCGAG-3′ (antisense). As a control, siRNA to GFP (Dharmacon, http://www.dharmacon.com) was used. Cells were grown to 50% confluency in 6-well plates and transfected with 1 μg of siRNAs using siFECTamine (IC-Vec, United Kingdom) [46]. The transfection efficiency was determined to be >97% using siFECTamine labeled with Dioleoyl-sn-Glycero-3-Phosphoethanolamine-N-(Lissamine-Rhodamine-B-Sulfonyl) (IC-Vec) by flow cytometry and immunofluorescence analysis (unpublished data).

### Semi-quantitative RT-PCR.

For semi-quantitative RT-PCR after siRNA transfection, total RNA was extracted from HeLa cells using TRIzol reagent (Invitrogen) and treated with Amplification grade DNAseI (Invitrogen) before semi-quantitative RT-PCR analysis was performed using the SuperScriptTM III One-Step RT-PCR System (Invitrogen). Each reaction contained 0.2 μg of total RNA and the optimal number of cycles for linear amplification for tissue expression analysis. Primers were h-GAAP forward: 5′-GCCCCGAATTCTGCCATCATGGCTGACCCCG-3′ and reverse: 5′-GCCCCTCGAGTTACTTTTTATTAACTGCTTCCAGAAACC-3′; and actin forward: 5′-GTGGGGCGCCCCAGGCACCA-3′ and reverse 5′-CTCCTTAATGTCACGCACGATTTC-3′.

To determine the time of expression of VACV GAAP during infection, TK^−^143 cells were infected with 5 pfu/cell of CMLV with or without 40 μg/ml AraC. After 2, 5, or 22 h, total RNA was extracted from cells using the RNEasy Midi kit (Qiagen, http://www.qiagen.com) treated with Amplification Grade DNAseI (Invitrogen). Semi-quntitative RT-PCR analysis was performed as above. Primers were v-GAAP exp1, v-GAAP exp2, D8L-1 (5′-GTCTCTCTCAAATCGGAC), and D8L-2 (5′-GCAAAAAAAACACGATGA).

### Mitochondrial membrane potential.

Mitochondrial membrane potential was quantified by staining with TMRE (Molecular Probes, http://probes.invitrogen.com). Cells were loaded with TMRE by incubating cells in medium containing 0.2 μM TMRE for 30 min at 37 °C. TMRE fluorescence was acquired using flow cytometry through the FL-2 channel.

### Virulence assays of v-GAAP mutant viruses in mice.

For intranasal infections, groups of BALB/c mice (6–8 wk old) were inoculated under general anaesthesia with virus in 20 μl PBS. Each day, mice were weighed individually and monitored for signs of illness [27], and those suffering a severe infection or having lost 30% of their original body weight were sacrificed. To obtain BAL fluid, mice were killed and their lungs were inflated five times with 1.5 ml of 12 mM lidocaine and 5 mM EDTA in Earle's Balanced Salt Solution (Sigma) through a blunted 23-gauge needle inserted into the trachea. BAL fluids were centrifuged (2,000 rpm, 10 min) and the cells were re-suspended in erythrocyte lysis buffer (0.829% NH_4_Cl, 0.1% KHCO_3_, 0.0372% Na_2_EDTA, [pH 7.2]) for 5 min at RT. Cells were washed with RPMI 1640 medium (Gibco) and cell viability was assessed using trypan blue exclusion.

### Statistical analysis.

The Student's *t*-test (two-tailed) was used to test the significance of the results.

## Supporting Information

Figure S1 Protein Trafficking to the Cell Surface Is Not Affected by GAAPs U2OS-neo, U2OS-v-GAAP, or U2OS-h-GAAP cells were transfected with a plasmid encoding vesicular stomatitis virus glycoprotein G fused to GFP and were fixed for analysis by fluorescence microscopy 4, 7, and 24 h post transfection.
Scale bars, 20 μm.
(584 KB PPT)

Figure S2 Down-Regulation of Human GAAP Induces Loss of Mitochondrial Membrane Potential HeLa cells, mock-transfected or transfected with h-GAAP siRNAs 1–3 for 56 h, were loaded with the mitochondrial membrane potential sensor TMRE. Loss of mitochondrial membrane potential was measured as a decrease in TMRE fluorescence by two-color flow cytometry (cell population labeled M1).
(76 KB PPT)

### Accession Numbers

The Genbank (http://www.ncbi.nlm.nih.gov/Genbank) accession numbers for the proteins discussed in this paper are A. thaliana GAAP (NP_193209), CMLV strain CM-S GAAP (AAG37461), dog GAAP (XP531662), D. melanogaster GAAP (Q9V6H6); h-GAAP (CGI-119, AAD34114; S1R protein, AAF14868; and z-protein, AF182041), mouse GAAP (Q9DA39), orangutan GAAP (Q5R8M0), VACV Evans GAAP (AY690669), VACV Lister GAAP (AY699305), VACV USSR GAAP (AY699306), X. laevis GAAP (AAH90219), and zebrafish GAAP (Q5TZH2).

## Abbreviations

aa: amino acid(s)
Ab: antibody
AraC: cytosine arabinoside
BAL: bronchoalveolar lavage
bp: base pair
cDNA: complementary DNA
CHX: cycloheximide
CMLV: camelpox virus
ER: endoplasmic reticulum
GAAP: Golgi anti-apoptotic protein
GFP: green fluorescent protein
HA: hemagglutinin
h-GAAP: human Golgi anti-apoptotic protein
mAb: monoclonal antibody
mRNA: messenger RNA
PARP: poly (adenosine diphosphate–ribose) polymerase
pfu: plaque-forming unit
p.i.: post infection
RT-PCR: reverse transcription PCR
SD: standard deviation
siRNA: small interfering RNA
TMRE: tetramethylrhodamine ethyl ester
TNF: tumour necrosis factor
VACV: vaccinia virus
v-GAAP: viral Golgi anti-apoptotic protein
