# Supplementary figures and images for: A New Inhibitor of Apoptosis from Vaccinia Virus and Eukaryotes
Source: PLoS Pathog. 2007 Feb 23;3(2):e17. doi: 10.1371/journal.ppat.0030017 (PMC1803007; doi:10.1371/journal.ppat.0030017)

## Slide 1
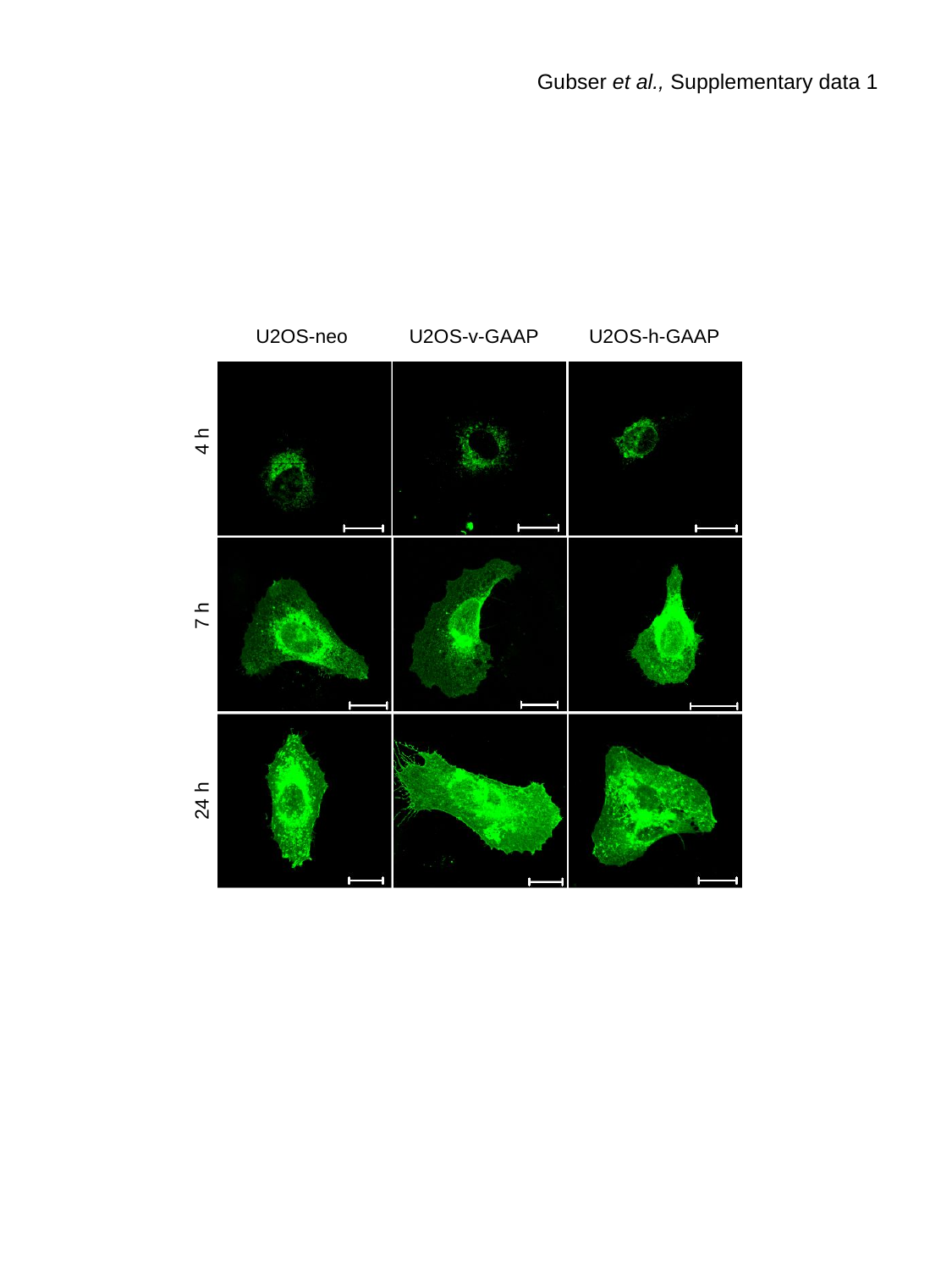

Gubser et al., Supplementary data 1
U2OS-neo
U2OS-v-GAAP
U2OS-h-GAAP
4 h
7 h
24 h

Supplement: Figure S1 — U2OS-neo, U2OS-v-GAAP, or U2OS-h-GAAP cells were transfected with a plasmid encoding vesicular stomatitis virus glycoprotein G fused to GFP and were fixed for analysis by fluorescence microscopy 4, 7, and 24 h post transfection. Scale bars, 20 μm. (584 KB PPT) [file ppat.0030017.sg001.ppt]
