# Supplementary material for: A New Inhibitor of Apoptosis from Vaccinia Virus and Eukaryotes
Source: PLoS Pathog. 2007 Feb 23;3(2):e17. doi: 10.1371/journal.ppat.0030017 (PMC1803007; doi:10.1371/journal.ppat.0030017)
Supplement: Figure S2 — HeLa cells, mock-transfected or transfected with h-GAAP siRNAs 1–3 for 56 h, were loaded with the mitochondrial membrane potential sensor TMRE. Loss of mitochondrial membrane potential was measured as a decrease in TMRE fluorescence by two-color flow cytometry (cell population labeled M1). (76 KB PPT) [file ppat.0030017.sg002.ppt]

## Slide 1
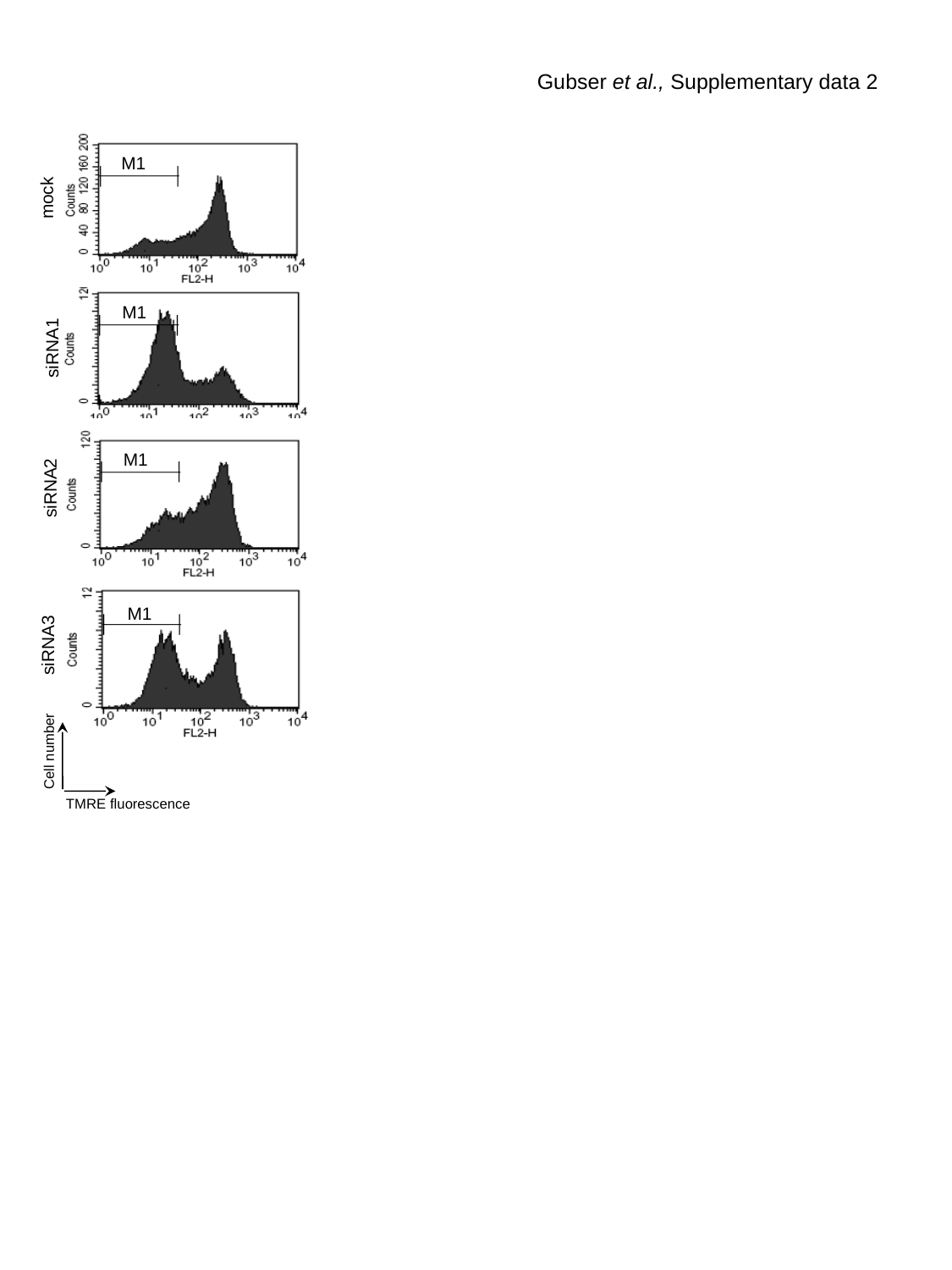

Gubser et al., Supplementary data 2
M1
mock
M1
siRNA1
M1
siRNA2
M1
siRNA3
Cell number
TMRE fluorescence
